# Outcome of intramedullary nailing treatment for intertrochanteric femoral fractures

**DOI:** 10.1186/s13018-019-1431-3

**Published:** 2019-11-12

**Authors:** Martin F. Hoffmann, Justin D. Khoriaty, Debra L. Sietsema, Clifford B. Jones

**Affiliations:** 1 Department of Surgery, Berufsgenossenschaftliches Universitätsklinikum Bergmannsheil Bochum, Bürkle-de-la-Camp-Platz 1, 44797 Bochum, Germany; 2 College of Human Medicine, Michigan State University, Grand Rapids, MI USA; 3 The CORE Institute, Phoenix, AZ USA

**Keywords:** Femur fracture, Intertrochanteric, Outcome, Nail, Nonunion, Smoking

## Abstract

**Introduction:**

The OTA/AO type 31 A3 intertrochanteric fracture has a transverse or reverse oblique fracture at the lesser trochanteric level, which accentuates the varus compressive stress in the region of the fracture and the implant. Intramedullary fixation using different types of nails is commonly preferred. The purpose of this study is to evaluate intertrochanteric femoral fractures with intramedullary nail treatment in regard to surgical procedure, complications, and clinical outcomes.

**Methods:**

From one level 1 trauma center, 216 consecutive adult intertrochanteric femoral fractures (OTA/AO type 31 A3) were retrospectively identified with intramedullary nail fixation from 2004 through 2013. Of these, 193 patients (58.5% female) met the inclusion criteria. The average age was 70 years (range 19–96 years).

**Results:**

Cephalomedullary nails were utilized in 176 and reconstruction nails in 17 patients. After the index procedure, 86% healed uneventfully. Nonunion development was observed in 6% and 5% had an unscheduled reoperation due to implant or fixation failure. Active smoking was reported in 16.6%. Current smokers had an increased nonunion risk compared to those who do not currently smoke (15.6% vs. 4.3%; *p* = 0.016). The femoral neck angle averaged 128.0° ± 5°. Fixation failure occurred in 11.1% of patients with a neck-shaft-angle < 125° compared to 2.6% (4/155) of patients with a neck-shaft angle ≥125° (*p* = 0.021). Patients treated with a reconstruction nail required a second surgical intervention in 23.5%, which was no different compared to 25.0% in the cephalomedullary group (*p* = 0.893). In the cephalomedullary group, 4.5% developed a nonunion compared to 23.5% in the reconstruction group (*p* = 0.002). Painful hardware led to hardware removal in 8.8%. All of them were treated with a cephalomedullary device (*p* = 0.180). During the last office visit, two-thirds of the patients reported no or only mild pain but most patients had reduced hip range of motion.

**Conclusion:**

Intramedullary nailing is a reliable surgical technique when performed with adequate reduction. Varus reduction with a neck-shaft angle < 125° resulted in an increase in fixation failures. Patient and implant factors affected nonunion formation. Smoking increased nonunion formation. Utilization of a cephalomedullary device reduced the nonunion rate, but had higher rates of painful prominent hardware compared to reconstruction nailing.

## Introduction

**Fig. 1:**
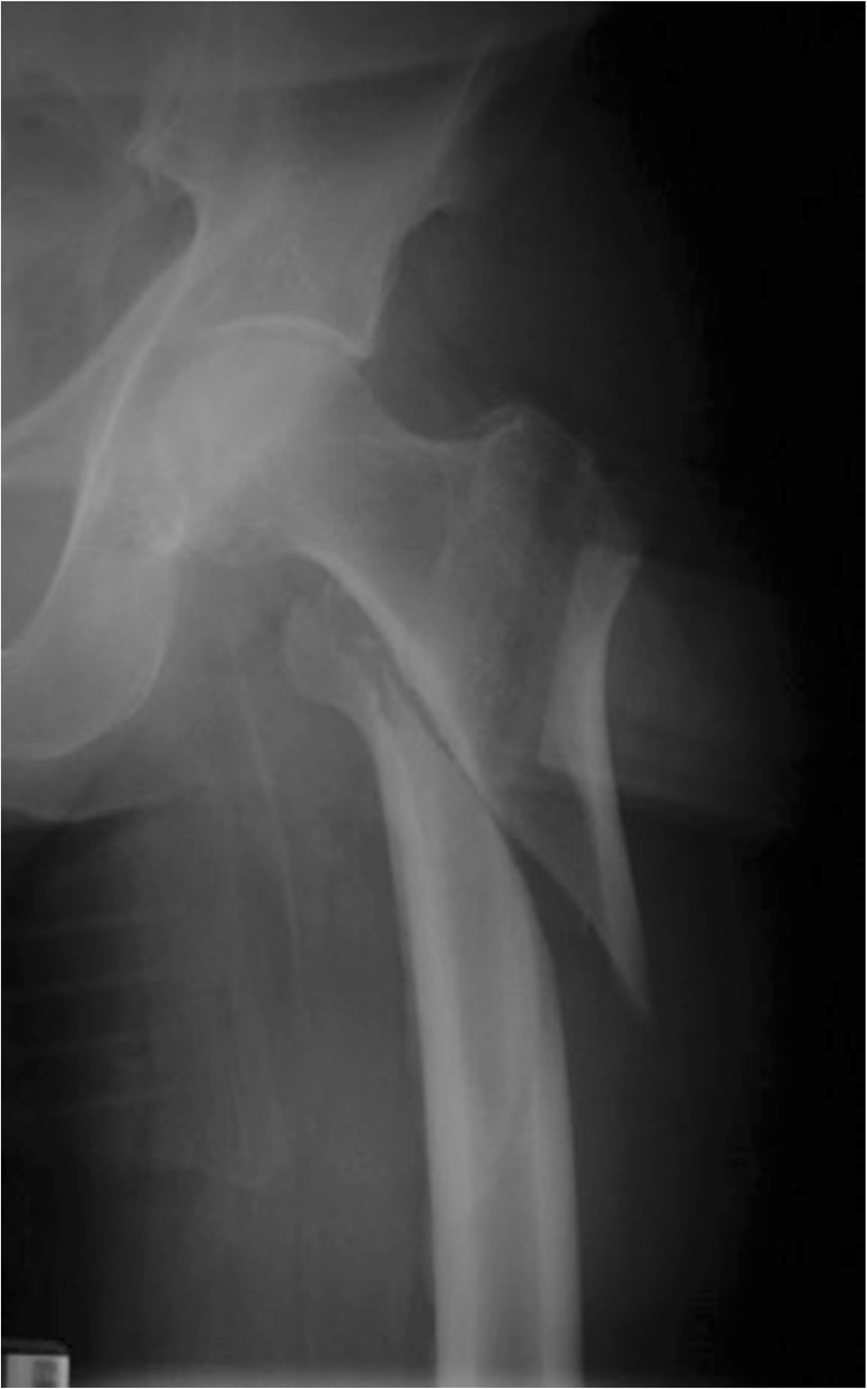
Reversed oblique femoral fracture type 31 A 3.1

**Fig. 2:**
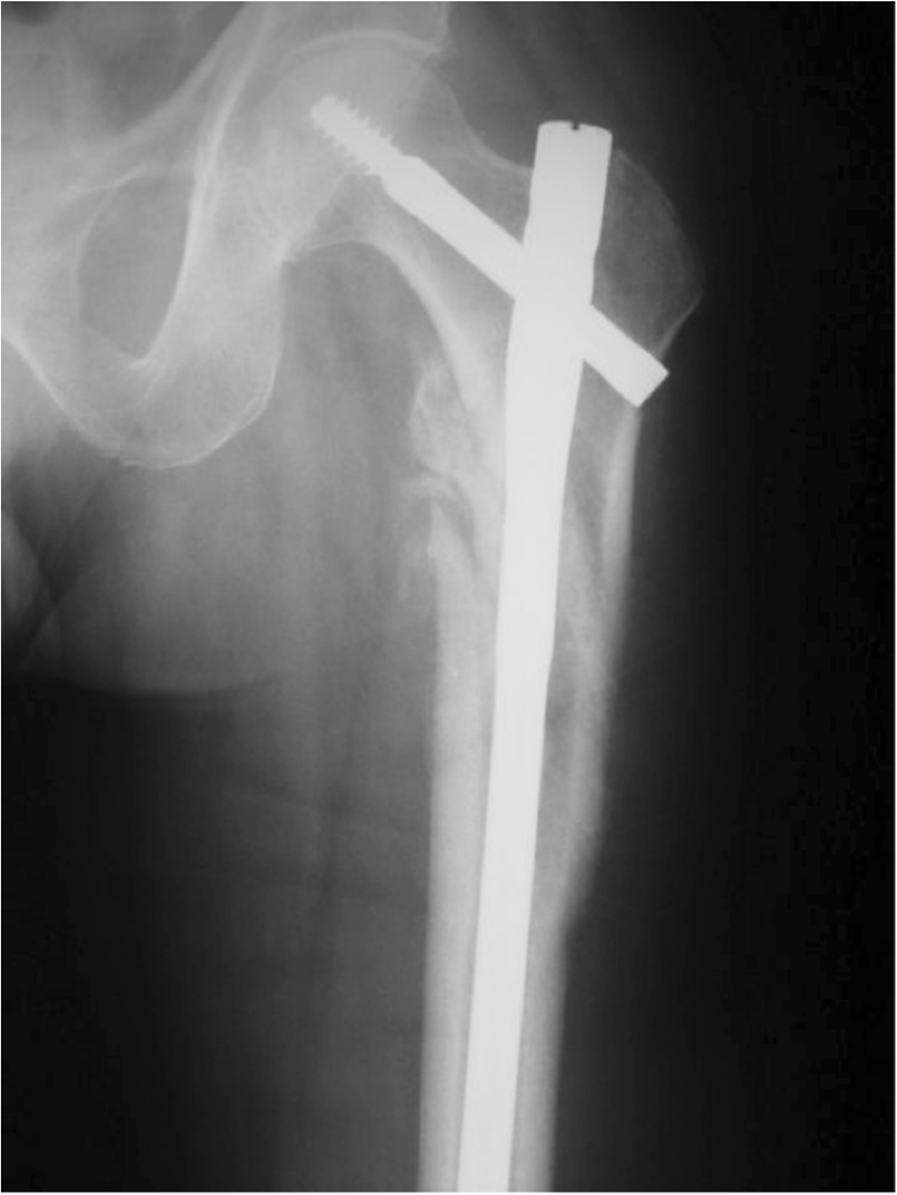
Intertrochanteric fracture treated with long cephalomedullary nail

Approximately 300,000 hip fractures occur in the USA each year [1, 2] with 40–45% being in the trochanteric region [3, 4]. In an effort to decrease the morbidity and the cost of treatment of these fractures, surgical techniques need to be optimized [5, 6]. The unique anatomy and the occurrence of high varying forces in the trochanteric region of the femur are challenging and demand sophisticated surgical treatment [7–9]. According to the Orthopedic Trauma Association (OTA)/Arbeitsgemeinschaft für Osteosynthesefragen (AO) pertrochanteric fractures run obliquely from the greater trochanter to the lesser trochanter (31 A1 and 31 A2) [10, 11]. This allows controlled impaction of the fracture site in compression hip screws and most intramedullary nailing systems [12]. Intertrochanteric fractures (type 31 A3 following the OTA/AO classification) [10, 11] have unique anatomic and mechanical characteristics and have been traditionally considered unstable [13, 14]. The OTA/AO type 31 A3 fractures are characterized by having a fracture line exiting the lateral femoral cortex distal to the vastus ridge [15]. Resulting in a fracture line that runs transverse or reversed oblique (Fig. 1) and leads to increased stress in the region of the fracture and the implant. Therefore, reduction, fixation, and maintenance of alignment until fracture healing are potential difficulties leading to high incidences of fixation failures with extramedullary devices [16], which are usually preferred for extracapsular hip fractures [17]. Related to the stability aspect intramedullary nailing has become popular for the treatment of unstable intertrochanteric hip fractures (Fig. 2) [18, 19].

There is a permanent confusion regarding the best treatment for fractures of the trochanteric region [13, 20, 21]. Many studies do not segregate precisely between pertrochanteric (type 31 A1 and 31 A2) and intertrochanteric (type 31 A3) fractures [11]. Two recent meta-analyses concluded that further studies are required to determine whether the intramedullary nail and what type of intramedullary nail is beneficial for intertrochanteric femur fractures [13, 17].

Therefore, the purpose of this study was to evaluate a series of intramedullary nail treatment of intertrochanteric femoral fractures (type 31 A3 according to the OTA/AO-classification) in regard to surgical procedure, complications, and clinical outcome.

## Patients and methods

This study was an Institutional Review Board approved retrospective cohort study of patients undergoing surgical treatment utilizing an intramedullary nail for intertrochanteric femur fractures (OTA/AO type 31 A3) between 2004 and 2013 in one level 1 trauma center by four fellowship-trained orthopedic trauma surgeons. The involved patients were collected from the clinic’s database based on a computer query of Current Procedural Terminology (CPT) codes for trochanteric fractures. Inclusion criteria were intertrochanteric femur fracture with long intramedullary nail (IMN) fixation and age equal to or older than 18 years. Exclusion criteria were intramedullary fixation with a short nail or utilization of an extramedullary implant [16, 22], follow up less than 6 months, metastatic disease, and insufficient medical record or radiographic data.

Two hundred and sixteen patients underwent surgical treatment for 216 intertrochanteric femur fractures during the study period. Twenty-three patients were excluded because of internal fixation with a short nail (3), intraoperative death (1), Girdlestone situation on the contralateral side (1), and loss to follow-up (18).

Each patient had two views of the initially injured femur. These were an anteroposterior (AP) view with the patient supine and a lateral view (LAT). Additional traction and oblique views or computed tomography scans (CT) with coronal and sagittal reconstructions were performed at the surgeon’s discretion for assessing fracture pattern and displacement.

Injury mechanism and potential contributing factors were recorded.

Fractures were classified according to the OTA/AO (Orthopedic Trauma Association/Arbeitsgemeinschaft Osteosynthese) system [10, 11].

Open or closed reduction and internal fixation of the intertrochanteric femur fracture was performed with the patient in the supine position on a radiolucent table with the injured leg draped freely or on a traction table. The operative approaches to the proximal femur were tailored to each patient based on the particular pattern of the injury, associated injuries, and soft tissue involvement. All intramedullary nails were distally locked following previous recommendations [23]. Four trauma fellowship-trained orthopedic surgeons performed the surgeries. All patients had initial postoperative radiographic imaging (AP, LAT) to confirm reduction quality and implant position. The reduction quality was addressed by measuring the neck-shaft-angle on the AP view and classifying the cortical step off in three categories (0 = no step off, 1 = <than 1 cortex width step off, and 2 = > 1 cortex width of step off) on the AP and lateral view. The effect of rotation on the neck-shaft-angle was addressed by comparing the initial radiograph to radiographs during follow-up [24].

Postoperatively, patients had antibiotic and deep vein prophylaxis. Patients were mobilized based upon the constellation of injuries and femur fracture pattern. Weight-bearing was allowed as tolerated. Formal physical therapy was instituted working on core strengthening, dynamic lumbar stabilization, range of motion, strengthening, conditioning, and gait training.

Patients were evaluated at regular and consistent intervals of 2 weeks, 6 weeks, 12 weeks, 6 months, 1 year, and 2 years or until bone healing was radiographically present. Complaints of pain with a visual analog scale and problems with ambulation were recorded. Clinical examination of incisional healing, motor exam, sensory exam, range of motion (ROM), and ambulation was performed. Radiographs consisting of AP and LAT views of the distal femur were obtained at each interval.

Complications were recorded concerning infection, union, hardware or fixation failure, and revision surgery. Infection was defined as either deep or superficial. Deep infections are defined as those that require operative treatment. Superficial infections are defined as those that are treated only with local antibiotics and wound care, and no operative treatment for the infection. Nonunion was defined as loss of fixation, not united radiographically, or continued pain at the fracture site. Further complications of leg length discrepancy, instability, and stiffness were recorded. Radiographic outcome was assessed with osseous healing, AP and sagittal alignment, and varus malalignment.

## Statistical analysis

Data was analyzed using PASW® 18. Descriptive statistics were completed. Chi-square and *t* tests were used to compare those that developed complications versus those that did not, such as demographic data, contributing factors, neck-shaft angle, and reduction quality. Pearson’s *r* or Spearman’s rho was used to analyze correlations between outcome, complication development, and other factors. If relationships were found, a predictive analysis using univariate or multivariate regression was conducted.

## Results

We identified 193 fractures in 193 patients with a mean age of 70.2 years. There were 80 (41.5%) males and 113 (58.5%) females with an average body mass index (BMI) of 26.7 kg/m^2^ (range 15.6–54.7 kg/m^2^). The mean length of hospital stay was 6 days (range 2–29 days) with a median of 5 days. The length of follow-up by regular office visits was 15.2 months (range 6–97) with a median of 11.5 months. There was an almost similar distribution between the right (90) and left (103) femur involved (46.6% and 53.4%, respectively).

Related to the advanced age of our study group, multiple comorbidities and risk factors were found (Table 1). Eighty of 193 patients (41.5%) had a history of smoking, and 32/193 patients (16.6%) were active smokers. Smoking increased the rate of nonunions. Patients, which admitted active or previous smoking, doubled their risk of forming a nonunion compared to patients that never smoked (8.8% vs. 4.4%; *p* = 0.226). Active smoking even triples/almost quadrupled the nonunion risk compared to those who do not currently smoke (15.6% vs. 4.3%; *p* = 0.016).

**Table 1:** Demographic data and contributing factors

Contributing factors	*n*	%
Total	193	100
Gender		
Male	80	41.5
Female	113	58.5
Age		
Years	70.2 (19–96)	
BMI		
kg/m^2^	26.7 (15.6–54.7)	
Diabetes	40	20.7
Cardiovascular or peripheral vascular disease	47	24.4
Respiratory disease	25	13.0
Smoking	32	16.6

High-energy mechanism (43; 22.3%) occurred in 28/80 (35%) male patients and in 15/113 (13%) female patients. With the majority (29/43; 67.4%) related to motorized vehicle accidents. 36/193 patients (18.7%) were classified as polytraumatized (ISS > 16) with 24/36 patients (66.7%) having additional injuries to the ipsilateral lower extremity. Open fractures (1.6%) occurred in one male patient and two female patients. Patients suffering from a low-energy fall were significantly older (76 years) than those with a high-energy trauma (50 years) (*p* < 0.001). Additionally, females (98/113; 86.7%) had a significantly increased risk for low-energy interochanteric fractures compared to males (52/80, 65.0%) (*p* < 0.001).

Fractures were classified according to the AO/OTA classification [10, 11]. All fractures were classified as 31 Type A3 fractures (Table 2).

**Table 2:** Fracture classification according to AO/OTA

Classification	31 A3.1	31 A3.2	31 A3.3
Number	30	39	124
Percentage	15.5	20.2	64.2

Different nailing systems and implant materials were used (Table 3). The average reduction of the femoral neck-shaft-angle was 128° ± 5° (range 113 to 140°). The reduction quality regarding cortical step-off showed no step off in 116/193 patients (60.1%), a step off ≤ to one cortical thickness in 45/193 (23.3%), and > 1 cortical thickness in 30/193 patients (15.5%). In the initial postoperative lateral views of two patients, no measurement of the step off was possible.

**Table 3:** Utilization of nail types

Implant type (manufacturer)	Frequency	%
Gamma 3 (Stryker)	5/193	2.6
TFN (Synthes)	4/193	2.1
M/DN (Zimmer)	9/193	4.7
ITST (Zimmer)	165/193	85.5
Sirus (Zimmer)	8/193	4.1
ZNN CMN (Zimmer)	2/193	1.0
Stainless Steel	174/193	90.2
Reconstruction nail	17/193	8.8

One hundred sixty-five of 193 fractures (85.5%) healed after the index procedure. Twelve of 193 (6.2%) developed a nonunion with additional 6 fractures (3.1%) resulting in a malunion. Nonunion development was not related to age (72.6 vs. 70.8 years, respectively; *p* = 0.657). Nine of 193 patients (4.7%) underwent a reoperation due to hardware or fixation failure with additional 2 patients (1.0%) diagnosed with avascular necrosis of the femoral head (AVFH) which were converted into total hip arthroplasties. In total, 13 patients (6.7%) had to be treated by hemi or total hip arthroplasty. Fixation failure was not related to patient age (66.6 vs. 71.3 years, respectively; *p* = 0.132). Hardware/fixation failure was not related to the type of nail, implant material, or AO/OTA classification, but fixation failure was related to neck-shaft-angle reduction quality. Fixation failure occurred in 11.1% (4/36) of patients with a neck-shaft-angle < 125° compared to 2.6% (4/155) of patients with a neck-shaft-angle ≥ 125° (*p* = 0.021). Nonunion formation was significantly greater in patients with an initial neck-shaft angle between 130°–134° compared to those with an angle > 134° (*p* = 0.042). For additional information please see Table 4.

**Table 4:** Complications related to neck-shaft-angle

Reduction quality	Total	< 125°	125°–129°	130°–134°	≥ 135°
No secondary surgery	145 (75.1%)	24 (16.6%)	63 (43.4%)	44 (30.3%)	13 (9.0%)
Secondary surgery	48 (24.9%)	12 (25.0%)	17 (34.7%)	17 (34.7%)	1 (2.1%)
Fixation failure	8	4 (50.0%)	3 (37.5%)	0 (0.0%)	1 (12.5%)
Nonunion	12	1 (8.3%)	3 (25.0%)	7 (58.3%)	0 (0.0%)
Infection/hematoma	2	0 (0.0%)	1 (50.0%)	1 (50.0%)	0 (0.0%)
Painful hardware	17	4 (23.5%)	7 (41.2%)	6 (35.3%)	0 (0.0%)
AVN of the femoral head	2	1 (50.0%)	1 (50.0%)	0 (0.0%)	0 (0.0%)
Malunion	6	2 (33.3%)	2 (33.3%)	2 (33.3%)	0 (0.0%)
Heterotopic ossifications	1	0 (0.0%)	0 (0.0%)	1 (100.0%)	0 (0.0%)

Comparing the implant type, 23.5% of patients treated with a straight nail required a second surgical intervention, which is no difference to 25.0% of the patients with cephalomedullary nails undergoing reoperation (*p* = 0.893), but all reoperations (100%) in the centromedullary group were for nonunion treatment. In the cephalomedullary group, 8/176 (4.5%) developed a nonunion compared to 4/17 (23.5%) in the centromedullary group (*p* = 0.002).

In contrast hereto, 17/193 patients (8.8%) complained about painful hardware leading to hardware removal. All of them were treated with a cephalomedullary device (*p* = 0.180).

### Clinical outcome

Despite the fact that weight-bearing as tolerated was allowed postoperatively, time to full weight-bearing was immediate to 52 weeks. The mean time was 5 weeks with a median of 2 weeks. Overall clinical alignment was stated as anatomic in 184/193 patients (95.3%) with 6 (3.1%) being in varus and 3 (1.6%) being in valgus. A leg length discrepancy was noted in 26/193 (13.5%), but only in patients with cephalomedullary devices. Fifty-six percent of the patients (109/193) reported no pain during the last office visit, leaving 84 (43.5%) with a persistent mean pain level of 3.6 (range 1–8/10). Still, thereof 58% had low pain levels resulting in a median pain level of 3.

Range of motion was addressed by physical exam. For range of motion, hip flexion at the final visit averaged 109° (40°–130°) with internal rotation 20° (0°–45°) and external rotation 33° (5°–70°).

## Discussion

The incidence of proximal femoral fractures is continually increasing due to the changes in population demographics. Especially for elderly patients, regaining pre-fracture ambulatory function and autonomy is crucial. Intramedullary and extramedullary fixations are the primary options for intra- and extracapsular fractures. Despite the superiority of the sliding hip screw compared to intramedullary nails for the treatment of extracapsular femur fractures [17], there is a permanent confusion regarding the best treatment for fractures of the trochanteric region [13, 20] with an increasing body of evidence suggesting that pertrochanteric fractures with subtrochanteric extension and intertrochanteric fractures are best treated with the use of an intramedullary long nail [5, 15, 25, 26]. Compared with extramedullary dynamic hip screw (DHS) fixation, intramedullary nail fixation confers a short-term advantage of early weight-bearing [27] especially in unstable per-/intertrochanteric fractures involving the posteromedial wall or lesser trochanter. In patients with such fractures treated with the DHS, weight bearing is delayed until bone union, so as to minimize the collapse of the fixation [28].

Additionally, many studies do not segregate precisely between pertrochanteric (type 31 A1 and 31 A2) and intertrochanteric (type 31 A3) fractures [11]. But especially the transverse or reversed oblique fracture line and the specific biomechanics of intertrochanteric fractures lead to difficulties of fixation and maintenance of alignment resulting in high incidences of fixation failures with extramedullary devices [16, 22].

We could show that these fracture types are reliably treated with intramedullary devices resulting in an 85.5% healing rate after the index procedure. Comparing our reoperation rate we found similar numbers in the literature [22, 29] but most studies do not report the effect of painful hardware which led to an almost 9% rate of hardware or partial hardware removal. Hou et al. found in a pertrochanteric study that the most common complication was pain secondary to lateral migration of the helical blade due to fracture side collapse [5]. Our study supports this finding. All patients that underwent hardware removal were treated with a cephalomedullary nail that allows sliding of the hip screw.

There is one clinical report of intramedullary hip nailing regarding the clinical significance of the lesser trochanteric fragment which differentiates AO/OTA 31 A3.1 and A3.2 from 31 A3.3 [12]. They could show that the union time was significantly prolonged in 31-A3.3 type fractures compared with the union time of 31-A3.1 and 31-A3.2 type fractures, leading to the conclusion that the lesser trochanteric fragment seems to play an important role in the stability after intramedullary nailing of intertrochanteric fractures [12]. In our study hardware or fixation failure was not related to the type of nail, implant material or AO/OTA classification, but a neck-shaft angle of < 125° led to a significant increase in fixation failure. The influence of varus malreduction for femur neck fractures and trochanteric fractures was described previously. A more varus reduction has been associated with a higher cut-out rate after SHS fixation [30]. A more valgus reduction seems to be beneficial for screw positioning resulting in stable fixation of the femoral head and neck [31]. Kashigar could also show a significant association between a more varus reduction and cut-out for cephalomedullary nailing [32]. Additionally, nonunion formation was reduced in patients with a postoperative neck-shaft angle > 134°. Regarding nonunion formation, the implant seems to influence bone healing. We found a significantly greater risk for nonunion formation in patients treated with a reconstruction nail compared to those treated with a cephallomedullary device.

The incidence of AVFH in a recent review was calculated 0.95% within the first year of follow-up, and with a minimum 2-year follow-up it was 1.37% [33]. In our study, two patients (1.04%) developed an avascular necrosis of the femoral head after nailing. The most probable cause appears to be a disruption of the extraosseous arterial blood supply to the femoral head [33]. Additionally, high-energy trauma with fracture comminution and displacement were suggested as risk factors, which are common in intertrochanteric fractures. In our study, total hip replacement was the mainstay of treatment.

Additionally to our finding of technique and implant-related factors for surgical outcome, patient-related factors influence bone healing. The deleterious effects of cigarette smoking on multiple organ systems and especially the different aspects of the musculoskeletal system have been demonstrated [34, 35]. The reduction of vascularization due to nicotine has previously been shown in a rabbit model [36]. Additionally, several studies showed the effect of smoking regarding delayed bone healing and nonunion formation [37–40]. Previous studies focused on cortical bone [37, 40–42]. This study supports previous findings and adds new information regarding spongy bone. In contrast to the Castillo study, reformed smokers in our study population returned to baseline risk to form a nonunion. This may be due to the fact that all office notes were checked for smoking and patients that were noted at least one time as current smokers during the entire follow up, were counted as current smokers [43].

We must admit limitations of our study including the possibility of surgeon bias towards the use of either a straight or cephallomedullary nail in certain fracture patterns, which remains hidden in our retrospective study design. Despite the fact that long nails seem to offer no clinical advantage compared to standard nails for the treatment of 31 A3 type fractures except a reduced rate of secondary femur fracture [22, 44, 45], we included only long nails. This is in accordance with the recently published algorithm for the treatment of per-/intertrochanteric fractures of the hip [19]. The importance of tip apex distance in proximal femoral fractures undergoing nailing has been addressed recently [46]. We acknowledge the limitation due to the fact that we did not measure the tip apex distance related to the fact that a useful tip apex distance does not exist for reconstruction nails. The strength of our study is based on its single-center design with 193 included patients and the strict differentiation between pertrochanteric and intertrochanteric (AO/OTA type 31 A3) fractures.

## Conclusion

Intramedullary nailing is a reliable surgical technique, when performed with adequate reduction. Varus reduction with a neck-shaft angle < 125° resulted in a significant increase in fixation failures. Patient and implant factors affected nonunion formation. Smoking significantly increased nonunion formation. The utilization of a cephalomedullary device significantly reduced the nonunion rate, but had higher rates of painful prominent hardware compared to reconstruction nailing.

## Data Availability

The datasets generated and analyzed during the current study are available from the corresponding author on reasonable request

## Abbreviations

AO: Arbeitsgemeinschaft osteosynthese
AP: Anteroposterior
AVFH: Avascular necrosis of the femoral head
BMI: Body mass index
CPT: Current procedural terminology
CT: Computed tomography
DHS: Dynamic hip screw
IMN: Intramedullary nail
ISS: Injury Severity Score
LAT: Lateral
OTA: Orthopedic Trauma Association
ROM: Range of motion
